# Cardiorespiratory modulation of cerebrospinal fluid flow in the presence of spinal subarachnoid space obstruction

**DOI:** 10.1186/s12987-026-00823-4

**Published:** 2026-06-09

**Authors:** Joel A. Berliner, Zac W. Penprase, Donald E. Ogolo, Shinuo Liu, Lynne E. Bilston, Marcus A. Stoodley, Sarah J. Hemley

**Affiliations:** 1 Macquarie Medical School, Faculty of Medicine, Health, and Human Sciences, Macquarie University, Sydney, NSW Australia; 2 School of Biomedical Engineering, Faculty of Engineering, University of New South Wales, Sydney, NSW Australia

**Keywords:** Cerebrospinal fluid, Spinal cord injury, Syringomyelia, Arachnoiditis, Hypertension, Tachycardia, Respiration

## Abstract

**Background:**

Traumatic injury to the spinal cord disrupts cerebrospinal fluid (CSF) flow in the spinal subarachnoid space (SAS) and can precipitate cystic accumulation of fluid within the tissue – syringomyelia. The dynamic processes that lead to syringomyelia from an obstruction to CSF flow are not clearly defined. Investigations into CSF circulation have revealed that physiological variables, including respiration, heart rate, and blood pressure, influence CSF flow. Spinal cord injury affects each of these variables, however it is unknown how these variables influence CSF flow when the SAS is obstructed.

**Methods:**

We examined the separate effects of respiratory pressure (spontaneous breathing and positive pressure ventilation), heart rate (tachycardia), and blood pressure (hypertension) on CSF flow in a rodent model of SAS obstruction. A C7 – T1 laminectomy was performed in male Sprague-Dawley rats, followed by a suture tied around the spinal dura to obstruct CSF flow. SAS and intraparenchymal hydrodynamics of intracisternally-infused fluorescent tracers were investigated through real-time intraoperative and epifluorescence imaging, respectively.

**Results:**

A SAS obstruction significantly reduced spinal CSF flow. In the presence of SAS obstruction, tachycardia may initially reduce spinal CSF flow whilst hypertension reduces CSF influx above the obstruction. In the brain, both hypertension and spontaneous breathing increased CSF tracer influx. SAS obstruction reduced tracer influx into the brain in spontaneously breathing animals only.

**Conclusions:**

Acute changes to respiratory pressure, heart rate, and blood pressure have spatial- and time-dependent effects on CSF flow in the spinal cord and brain. Correcting for cardiorespiratory dysfunction in people living with SCI may disrupt the processes leading to syringomyelia, but further investigations are required.

**Supplementary Information:**

The online version contains supplementary material available at 10.1186/s12987-026-00823-4.

## Introduction

SCI can lead to inflammation and scarring (arachnoiditis) surrounding the spinal cord. The fibrotic thickening and arachnoid adhesions can obstruct the normal flow of CSF in the SAS [1, 2]. This is considered an important factor in the development of post-traumatic syringomyelia (PTS), which causes additional neurological impairment and pain in SCI patients [3]. The processes and predictors of PTS are poorly understood, with onset occurring anywhere from one month to 45 years post-injury [4] and affecting up to one-third of people living with SCI [5].

People living with SCI are often affected by major sensory, motor, and autonomic dysfunction. The physiological variables of respiration, blood pressure, and heart rate can become dysregulated [6]. These variables have been suggested to influence the normal movement of CSF along subarachnoid and perivascular pathways. Magnetic resonance imaging (MRI) and computational modelling studies in the human brain have shown that respiration and the arterial pulse wave drive CSF flow in the SAS [7–10]. However, in rodents, in vivo CSF flow studies have reported distinct findings between cranial and spinal compartments, with hypertension reducing overall flow along brain periarterial spaces [11] and intrathoracic pressure changes affecting subarachnoid and interstitial flow in the spine [12].

As cardiorespiratory variables influence the normal spinal subarachnoid and intraparenchymal flow of CSF, we aimed to assess their influence in the presence of a SAS obstruction. Experimental manipulation of respiratory pressure, blood pressure, and heart rate were independently achieved in a rodent model of SAS obstruction, created by extradural constriction, with SAS and intraparenchymal distribution of CSF movement quantified.

## Methods

### Animals

All experiments reported in this study were approved by the Macquarie University Animal Ethics Committee (Protocol No. 2019/021). In all eight experimental groups, a minimum of 10 animals were operated on, with a statistical requirement of 5 animals per group. Due to the complexity of the experimental procedures, it was expected that some animals would need to be excluded due to failure of the obstruction or CSF leak. Fifty-four adult male Sprague-Dawley rats weighing 366.1 ± 35.4 g and aged 11–13 weeks old were successfully used to study CSF tracer circulation in the SAS in vivo and deposition into the spinal cord and brain ex vivo. CSF circulation was examined after animals were exposed to changes in respiratory pressure, heart rate, and blood pressure. The cardiorespiratory variables being modulated were randomly assigned to each rat. Male rats were used in this study as SCI disproportionately affects males, accounting for approximately 80% of cases [13]. Sex was not considered as a biological variable in this study. The findings of this study are expected to be relevant to more than one sex.

### Surgical preparation

All surgical procedures were performed under inhalation anesthesia, with induction at 5% isoflurane in 1 L/min oxygen and maintenance at 2–3% isoflurane in 0.2 L/min oxygen. A pulse oximeter continuously monitored heart rate, oxygen (O_2_) saturation, and respiratory rate, whilst a rectal thermometer and heat mat were used to maintain core body temperature within normal range.

At commencement of surgery, surgical sites were shaved, and the animal was placed supine under a OPMI Pentero 800 microscope (Carl Zeiss, Oberkochen, Germany). To expose the femoral neurovascular bundle for cannulation, a right transverse inguinal incision was made. Polyethylene catheters were used to establish arterial and central venous lines, to measure mean arterial pressure, and deliver fluids and drugs, respectively. A suprasternal midline incision was then made to expose the trachea for endotracheal tube insertion. Tracheal tubing was connected to a small animal ventilator (UgoBasile, Gemonio, Italy) and set to 60 breaths/min, with tidal volume set at ~ 7.1 mL/kg based on average tidal volume in awake animals [14]. The egress tubing was connected to a capnometer (Capstar-100, CWE Inc., Ardmore, PA, USA) for continuous monitoring of end-tidal carbon dioxide (CO_2_) concentration. Physiological variables, CO_2_, blood pressure, and heart rate (derived from blood pressure) were digitized (1401, Cambridge Electronic Design (CED), Cambridge, UK) and recorded using Spike2 software (CED, Cambridge, UK). For the cohort of rats where heart rate was manipulated, a customized atrial pacing wire was inserted into the left external jugular vein and advanced until the tip was situated just proximal to the sinoatrial node [15]. The pacing wire was controlled by a pulse stimulator (A-M Systems Inc, model 2100). After the animal was repositioned prone, a midline dorsal incision and underlying muscle dissection was performed to expose the atlanto-occipital membrane and dorsal bony elements of C2 to T2. The spinal cord was exposed by a wide laminectomy to C7 and T1 vertebrae. For animals with a CSF obstruction, a 6 − 0 polypropylene monofilament suture (Ethicon, Johnson and Johnson Medical Pacific Pty Ltd, Sydney, Australia) was passed around the spinal cord (outside the dura) and tied with a reef knot. Occlusion of the dorsal vein was used as a visual indication that the SAS was obstructed. Normal SAS animals did not receive the extradural suture and CSF flow remained unobstructed.

### Manipulation of physiological parameters

#### Respiration

All rats were ventilated through the inserted endotracheal tube up until the point of CSF tracer injection. To examine the effects of respiration on CSF flow, animals were either allowed to breathe spontaneously (normal SAS, *n* = 8, SAS obstruction, *n* = 6), generating alternating negative and positive intrathoracic pressure, or they continued to be positive-pressure ventilated (normal SAS, *n* = 7, SAS obstruction, *n* = 6). Ventilated animals were administered with a neuromuscular blockade (pancuronium bromide, 0.8 mg), preventing diaphragmatic breathing and ensuring that only positive intrathoracic pressure was generated. Bradypnea was observed in spontaneously breathing rats, with resultant arterial blood CO_2_ retention (hypercapnia) and respiratory acidosis. Therefore, positive-pressure ventilated animals were set with a respiratory rate of 50–52 breaths/min (reduced from the initial 60 breaths/min) matching that of their spontaneous breathing counterparts (48–52 breaths/min). The positive-pressure ventilated animals were normotensive and normocardic and also used as a control for the blood pressure and heart rate experiments. Weight, heart rate, and mean arterial pressure (MAP) were kept constant between spontaneous breathing and positive-pressure ventilated animals (Supplementary Fig. 1).

#### Blood pressure

To examine the effects of blood pressure on CSF flow, hypertensive rats were compared to the normotensive animals. MAP was raised to a target of 140 mmHg in hypertensive rats (121.5 ± 17.5 mmHg in normal SAS animals (*n* = 6), 131.9 ± 4.1 mmHg in SAS obstruction animals (*n* = 6)) compared to the MAP of ~ 70 mmHg in normotensive animals (71.2 ± 8.0 mmHg in normal SAS animals (*n* = 7), 63.4 ± 8.9 mmHg in SAS obstruction animals (*n* = 6)) (a ~ 40 mmHg increase from baseline, or approximately double that of normotensive counterparts). This was achieved by infusion of phenylephrine (200 µg/kg/min), a selective α1-adrenergic receptor agonist. All hypertensive and normotensive animals were positive-pressure ventilated to relative hypercapnic levels. Weight and ventilation were kept constant between hypertensive and normotensive animals; however, heart rate was slightly increased in hypertension (Supplementary Fig. 1).

#### Heart rate

To examine the effect of heart rate on CSF flow, tachycardic rats were compared to the normocardic animals. The pacing wire connected to a pulse stimulator delivered a square pulse (duration of 2 ms and an amplitude of approximately 1.0 V) to the sinoatrial node to generate a heart rate of ~ 500 beats/min (bpm) to induce tachycardia (499.2 ± 2.9 bpm in normal SAS animals (*n* = 8), 502.4 ± 10.0 bpm in SAS obstruction animals (*n* = 5)). Heart rate for normocardic animals was ~ 330 bpm (326.2 ± 23.5 and 306.9 ± 24.7 bpm for normal SAS (*n* = 7) and SAS obstruction groups (*n* = 6), respectively). There was no appreciable change in blood pressure on pacing, even over prolonged periods (63.1 ± 8.8 mmHg compared to 67.6 mmHg ± 9.0 for normocardic animals). All tachycardic and normocardic animals were positive-pressure ventilated to a relative hypercapnic level. Weight, MAP, and ventilation were kept constant between tachycardic and normocardic animals (Supplementary Fig. 1).

### Arterial blood gas profile

Arterial blood pH, partial pressure of CO_2_, and partial pressure of O_2_ were measured immediately before and after CSF tracer imaging (Supplementary Fig. 2 and Supplementary Fig. 3) using a VetStat^®^ Electrolyte Blood Gas Analyser (Index Laboratories, Sydney, Australia).

### Procedures for investigation of fluid dynamics

CSF tracer movement was assessed using a 10 µL solution containing a mixture of the following two tracers: 5 µL indocyanine green (ICG, Verdye, Aschheim-Dornach, Germany) at 5 µg/µL and 5 µL Ovalbumin Alexa-Fluor^Ⓡ^-647 conjugate (OA-647: Life Technologies, Victoria, Australia) at 40 µg/µL. The ICG was detected in vivo using the inbuilt near-infrared filter on the surgical microscope, however, the instability of ICG limit its utility in ex vivo studies. All ex vivo analyses measured fluorescence of the dual-injected tracer, OA-647.

The cisterna magna cannula was constructed by attaching the tip of a 30G dental needle (T1-1DN-3022, Terumo, Sydney, Australia) to a 50 cm length of PVC polyethylene tubing (ID: 0.2 mm; OD: 0.5 mm) (Microtube Extrusions Pty. Ltd., North Rocks, NSW, Australia), ensuring that ~ 2 mm of the dental needle extended from the tubing. The other end of the tubing was then attached to a 30G Hamilton syringe (Hamilton Company, Nevada, USA) and connected to an Ultramicro Pump (World Precision Instruments, Florida, USA). The cisterna magna was cannulated through the exposed atlanto-occipital membrane using a single pass. A drop of cyanoacrylate glue (UHU GmbH and Co. KG, Buhl, Germany) was applied to the site, followed by a drop of sodium bicarbonate (0.08 g/mL in distilled water) to accelerate the curing time. At this point, the desired physiological variables were modulated to achieve the desired parameters for respiration, heart rate, and blood pressure for each experimental group.

### In vivo near-infrared imaging

Characterization of CSF movement in the cervicothoracic SAS was performed in vivo using near-infrared imaging of intracisternally injected ICG. Using the OPMI Pentero 800 microscope with its near-infrared camera function, the objective was positioned 30 cm above the surgical bench, zoom = 3.5×, and the field of view encompassing the craniocervical junction to T2 vertebra. The 10 µL tracer solution was then infused into the CSF circulation at a rate of 33 nL/s. Imaging commenced immediately following the tracer injection, recording the near-infrared and corresponding white light view simultaneously (at a rate of 25 frames/s). At 20 min from the start of tracer injection, the animal underwent transcardiac perfusion with heparinized 0.01 M phosphate buffered saline (PBS) followed by 4% paraformaldehyde (PFA) (Lancaster Synthesis, New Hampshire, USA).

### Ex vivo epifluorescence microscopy

Redistribution of intracisternally injected OA-647 tracer to the cervicothoracic spinal cord interstitium was quantified ex vivo. After perfusion-fixation, the neuraxis was harvested *en bloc* and post-fixed. Anterior (i), thalamic (ii), and, cerebellar (iii) brain regions, and spinal cord segments C2 to T4 were cryoprotected in 30% sucrose (in PBS), embedded in Optimal Cutting Temperature compound (Sigen, California, USA), frozen on dry ice, and stored at -80 °C. Coronal spinal cord and brain sections were cut on a cryostat (Leica CM1950, Leica Microsystems Pty Ltd, VIC, Australia) at 40 μm thickness and mounted onto glass slides (Superfrost™ Plus Adhesion Microscope Slides, New Erie Scientific LLC, USA). Sections were imaged at 20× magnification with a Zeiss Axio Imager fluorescence microscope (Carl Zeiss Microimaging GmbH, Germany). Identical exposure times were used for all images.

### Image processing and analysis

#### CSF tracer flow in the SAS

Intraoperative near-infrared fluorescence and white light videos were converted to TIFF Image Sequences using Adobe Photoshop 2023 (version 24.7, Adobe Systems Incorporated, California, USA) and opened as image stacks using ImageJ, v1.54 f. A region of interest for each spinal cord level (C2 – T2) was delineated using the white light image and then overlaid onto the matched IR800 image. Background signal was subtracted in each frame to give the integrated density (mean pixel value multiplied by area) at each region of interest from C2 to T2. Integrated density was measured every 30 s.

#### CSF tracer transport into the spinal cord and brain

To measure OA-647 influx in the brain and spinal cord, a region of interest that captured the parenchyma while excluding the meninges and spinal nerve roots was delineated using the free-hand drawing tool in ImageJ. Background signal was subtracted in each frame and the integrated density of intraparenchymal OA-647 was calculated for each brain region and spinal cord level. Three sections were analysed per spinal cord level (C2 to T4) and brain region (anterior (i), thalamic (ii), and cerebellar (iii) brain regions) and averaged.

### Statistics

Statistical comparisons of individual physiological variables were carried out using a one-way analysis of variance (ANOVA) and values were expressed as mean ± standard deviation, with adjustment for multiple comparisons conducted by Bonferroni’s *post hoc* tests. Fluorescence intensities (integrated densities) of the intracisternally-injected tracers were compared using two-way ANOVA, adjusted for multiple comparisons using Bonferroni’s *post hoc* tests. Values are expressed as mean ± SD. Technical replication is represented by *n* in the figure legends. A *p* value < 0.05 was considered statistically significant in all analyses. GraphPad Prism (v10.3.1, GraphPad Software Inc, California) was used to perform all statistical analyses and graph generation.

## Results

### Physiological factors did not alter spinal CSF flow in animals with a normal SAS

Fluorescence imaging of spinal levels C2 – T2 showed that respiratory pressure (spontaneous breathing with alternating positive and negative intrathoracic pressures), blood pressure (hypertension), and heart rate (tachycardia) had no effect on CSF tracer movement in animals with a normal SAS when compared to positive-pressure ventilated animals with normal blood pressure (normotension) and normal heart rate (normocardia) (Supplementary Fig. 4).

### SAS obstruction reduced spinal CSF flow

**Fig. 1:**
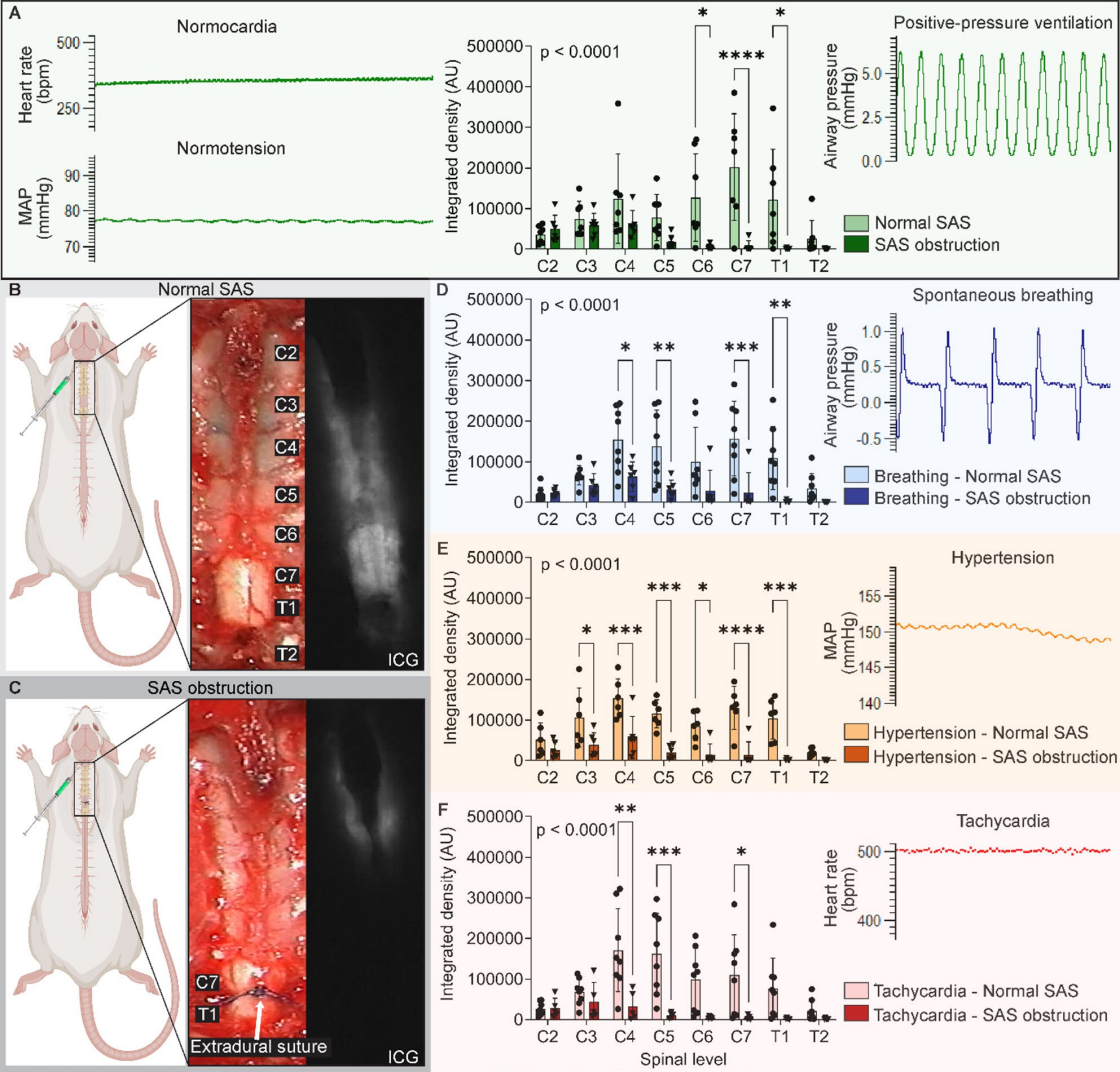
Subarachnoid space (SAS) obstruction reduced spinal cerebrospinal fluid (CSF) flow. Movement of the CSF tracer indocyanine green (ICG) was imaged in vivo using a near infrared filter on a surgical microscope. Graphs show fluorescence intensity at spinal levels 20 min following tracer injection. (**A**) Significantly higher fluorescence was recorded in normal SAS animals compared to SAS obstruction animals (*n* = 6, 7). These animals underwent positive-pressure ventilation, with no manipulation of heart rate (normocardia) or blood pressure (normotension). (**B**, **C**) Illustration of rat model of (**B**) normal SAS and (**C**) spinal SAS obstruction, intraoperative view of spinal column, laminectomy at C7 – T1, and (**C**) extradural suture tied to obstruct the SAS. Representative fluorescent images demonstrate ICG movement in the spinal SAS. In (**D**) spontaneously breathing animals (*n* = 6, 8), (**E**) hypertensive animals (*n* = 6), and (**F**) tachycardic animals (*n* = 5, 8), a SAS obstruction significantly reduced spinal CSF flow. The results in **(A**, **D** – **F)** are presented as mean integrated density ± SD, *p ≤ 0.05, **p ≤ 0.01, ***p ≤ 0.001, ****p ≤ 0.0001; two-way ANOVA + post hoc Bonferroni test

SAS obstruction significantly reduced the flow of CSF tracer (ICG) in the SAS compared to animals with a normal SAS (Fig. 1A, D – F; *p* < 0.0001).

### Tachycardia reduced spinal CSF flow in SAS obstruction animals, and this effect abated over time

**Fig. 2:**
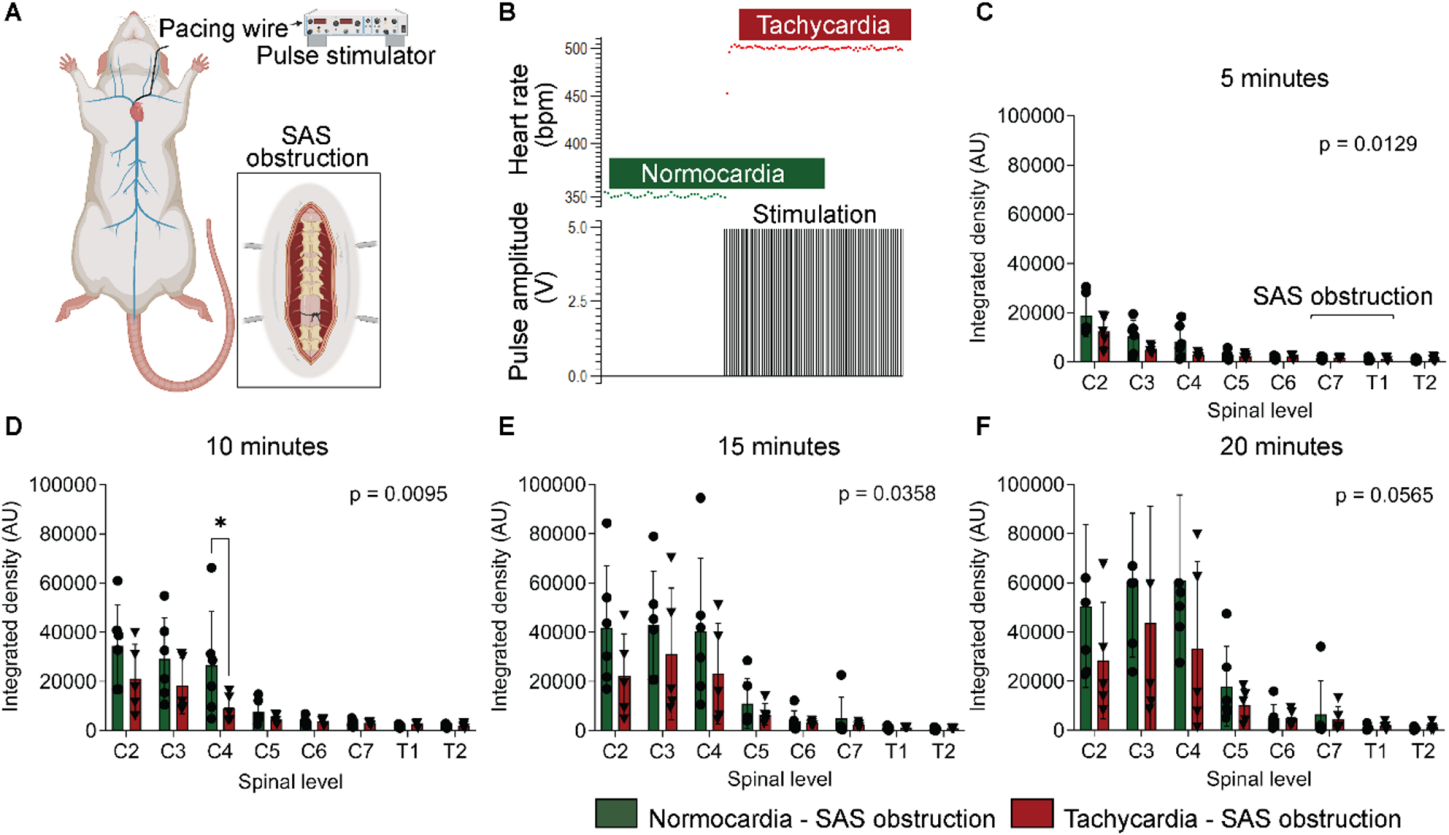
Tachycardia reduced cerebrospinal fluid (CSF) flow in the presence of a subarachnoid space (SAS) obstruction. (**A**) Illustration of SAS obstruction and atrial pacing to induce tachycardia. (**B**) Representative heart rate recording from an animal before (normocardia) and during (tachycardia) cardiac stimulation. In animals with a SAS obstruction, tachycardia significantly reduced fluoresence intensity in the spinal SAS at: (**C**) 5, (**D**) 10, and (**E**) 15 min following CSF tracer injection. (**F**) At the 20 min time-point there was no significant difference in CSF tracer movement between heart rate groups (normocardia, *n* = 6; tachycardia, *n* = 5). The results in (**C** – **F**) are presented as mean integrated density ± SD, *p ≤ 0.05; two-way ANOVA + post hoc Bonferroni test

Neither spontaneous breathing nor hypertension altered SAS CSF flow in animals with a SAS obstruction (Supplementary Fig. 5) when compared with the positive-pressure ventilated, normotensive animals. When heart rate was elevated with an atrial pacing wire (Fig. 2A, B) in SAS obstruction animals, spinal CSF flow was significantly lower compared to normocardic animals. The reduction in spinal CSF flow due to induced tachycardia lasted for 15 min from the commencement of tracer injection (Fig. 2C – E). After 15 min, this effect became non-significant (Fig. 2F).

### Hypertension and SAS obstruction reduced CSF tracer influx into the spinal cord

**Fig. 3:**
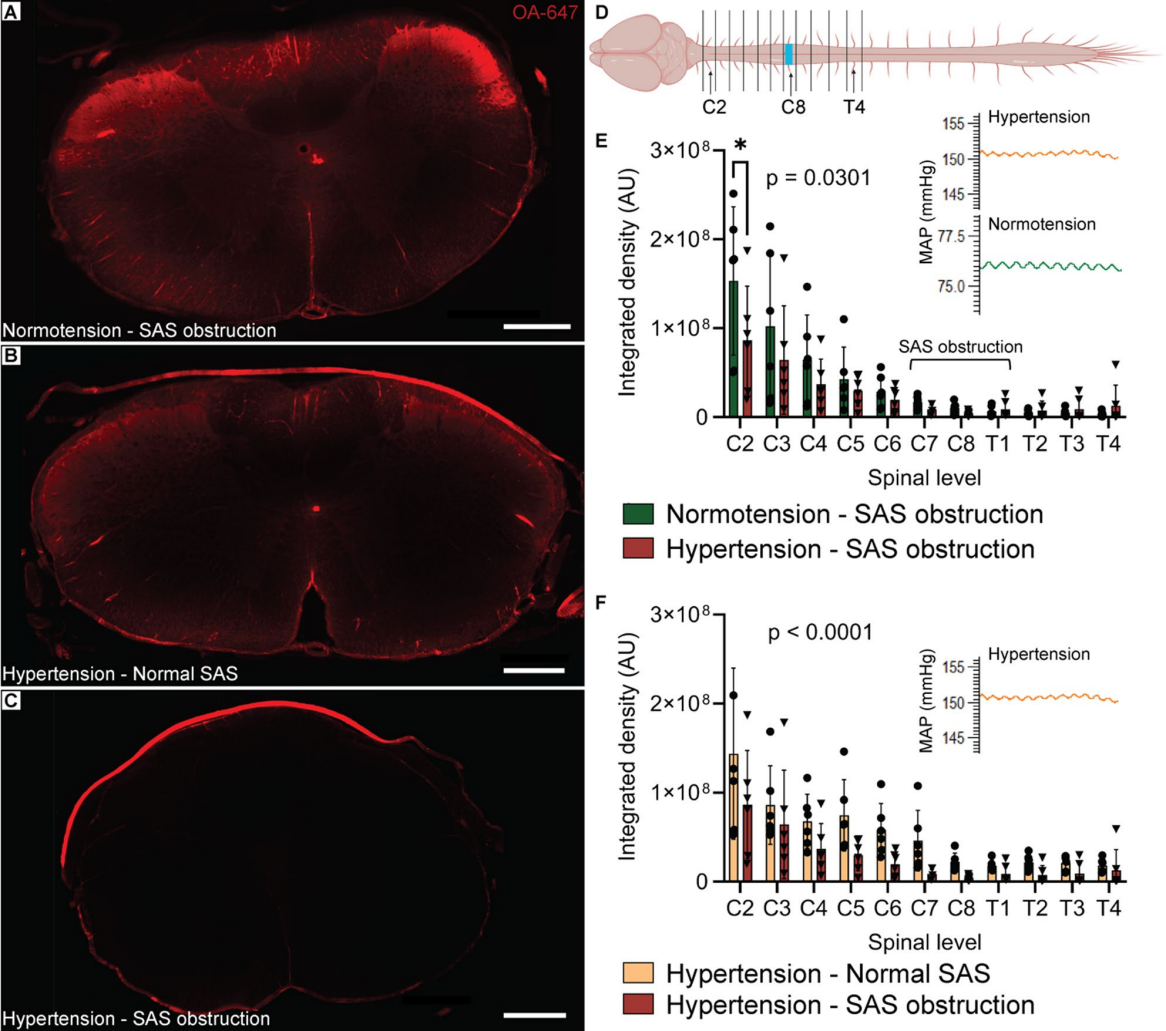
Hypertension and subarachnoid space (SAS) obstruction reduced cerebrospinal fluid (CSF) flow into the spinal cord. Coronal spinal cord sections at the C2 level showing the distribution of the CSF tracer ovalbumin Alexa Fluor-647 conjugate (OA-647) in the spinal cord in: (**A**) normotensive animals with a SAS obstruction (*n* = 6), (**B**) hypertensive animals with a normal SAS (*n* = 6), and (**C**) hypertensive animals with a SAS obstruction (*n* = 6). (**D**) Illustration depicting the segmentation of the spinal cord following transcardiac paraformaldehyde perfusion and fixation. Region of obstruction is indicated in blue at C8. Graphs in (**E** and **F**) show fluorescence intensity of spinal cord levels at 20 min following tracer injection. (**E**) In animals with a SAS obstruction, hypertension significantly reduced CSF tracer influx into the spinal cord above the obstruction. (**F**) In hypertensive animals, a SAS obstruction significantly reduced tracer influx into the spinal parenchyma. The results in (**E** and **F**) are presented as mean integrated density ± SD, *p ≤ 0.05; two-way ANOVA + post hoc Bonferroni test. Scale bars in (**A** – **C**) = 500 μm

Fluorescence imaging of spinal cord segments C2 – T4 (representative C2 micrographs, Fig. 3A – C) revealed a decrease in CSF tracer deposition in the cervical cord (above the obstruction) in hypertensive animals with SAS obstruction compared to normotensive animals with a SAS obstruction (Fig. 3E, *p* = 0.0301). *Post hoc* analysis revealed that hypertension significantly decreased tracer influx at spinal cord segment C2 (*p* < 0.05). In hypertensive animals, a SAS obstruction significantly decreased OA-647 influx to the spinal cord compared with animals with a normal SAS (Fig. 3F, *p* < 0.0001). Spontaneous breathing and tachycardia did not affect OA-647 influx to the cord interstitium in animals with a normal SAS or CSF obstruction (Supplementary Fig. 6).

### Hypertension and spontaneous breathing increased CSF tracer influx in the brain

**Fig. 4:**
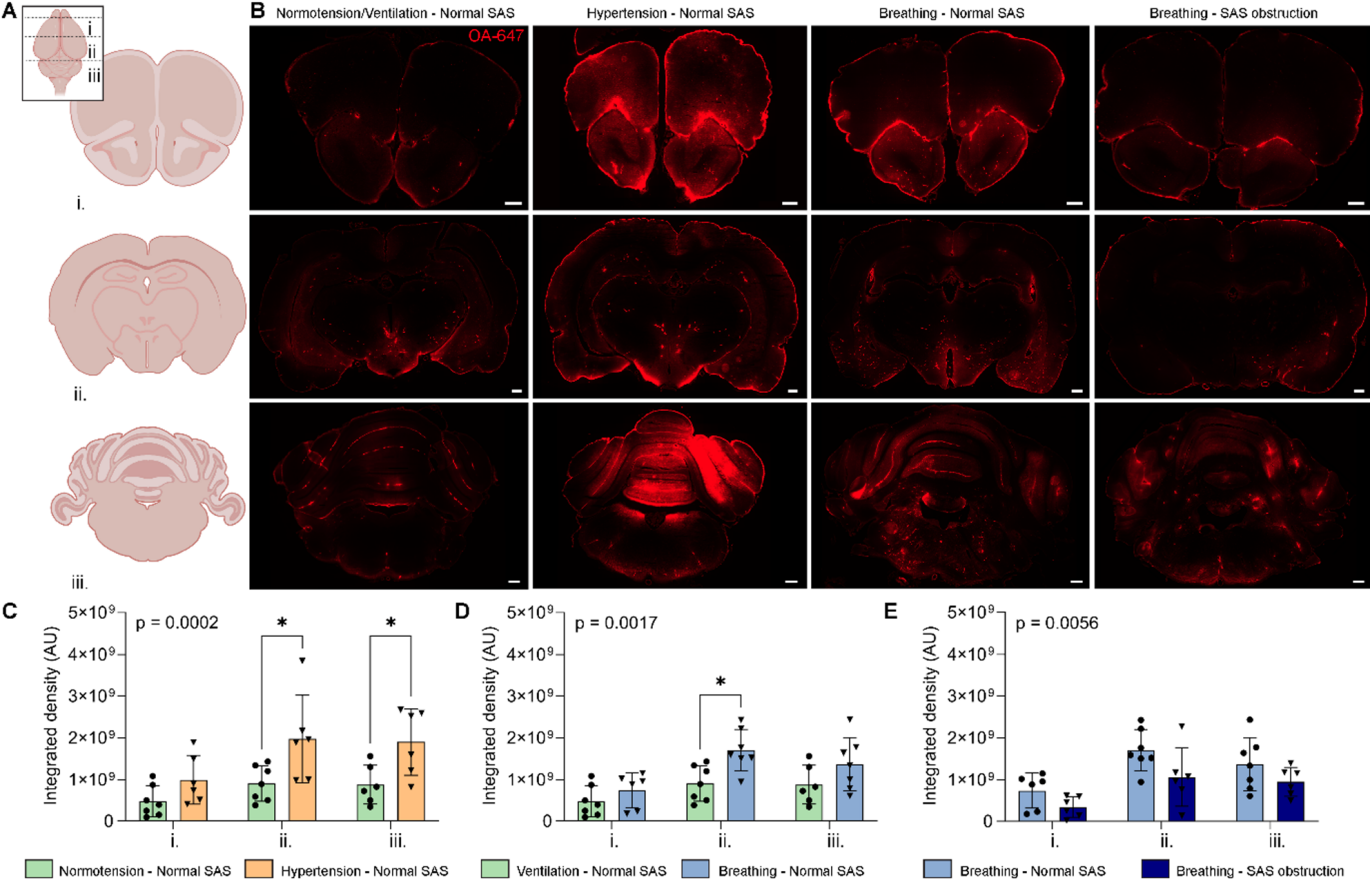
Hypertension and respiration altered cerebrospinal fluid (CSF) flow into the brain. (**A**) Illustration of coronal brain sections used to analyse the CSF tracer ovalbumin Alexa Fluor-647 conjugate (OA-647) deposition as a marker of CSF influx to the brain from the SAS. In each animal 3 replicates were imaged and analysed for each brain region. (**B**) Representative micrographs of brain regions (i, ii, and, iii) from animals with a normal SAS, and: normal physiological variables (normocardia and normotension) undergoing positive-pressure ventilation (*n* = 7), hypertension (*n* = 6), and breathing spontaneously (*n* = 7). Micrographs from spontaneously breathing animals with a SAS obstruction are also shown (*n* = 6). Graphs in (**C** – **E**) show fluorescence intensity of brain regions at 20 min following tracer injection. In animals with a normal SAS, (**C**) hypertension increased CSF tracer influx into the brain. (**D**) Spontaneous breathing also increased CSF tracer influx into the brain compared to positive-pressure ventilation. (**E**) In spontaneous breathing animals, a SAS obstruction significantly reduced tracer influx into the brain compared to a normal SAS. The results in (**C** – **E**) are presented as mean integrated density ± SD, *p ≤ 0.05; two-way ANOVA + post hoc Bonferroni test. Scale bars in (**B**) are 500 μm

Fluorescence imaging of rat brain (representative diagram and micrographs, Fig. 4A, B) revealed an increase in OA-647 tracer influx into the brain parenchyma in hypertensive animals with a normal SAS compared to their normotensive counterparts (Fig. 4C, *p* = 0.0002). *Post hoc* analysis revealed that hypertension significantly increased CSF influx into brain region (ii) (*p* < 0.05) and (iii) (*p* < 0.05). Similarly, tracer influx into the brain was greater in spontaneously breathing animals compared to animals that underwent positive-pressure ventilation (Fig. 4D, *p* = 0.0017), with the greatest increase localized to brain region (ii) (*p* < 0.05). Tachycardia did not alter CSF influx into the brain (Supplementary Fig. 7A).

### SAS obstruction reduced tracer influx into the brain in spontaneously breathing animals only

In spontaneously breathing animals, a SAS obstruction resulted in decreased OA-647 influx to the brain interstitium compared to animals with a normal SAS (Fig. 4E, *p* = 0.0056). Positive-pressure ventilation, hypertension, and tachycardia did not alter tracer deposition in the brain tissue of animals with SAS obstruction compared to animals with a normal SAS (Supplementary Fig. 7B – D). When the effect of modulating breathing, blood pressure, and heart rate was examined in animals with a SAS obstruction, these physiological variables had no effect on tracer deposition in the brain (Supplementary Fig. 6E – G).

## Discussion

This is the first in vivo study in rodents of the effects of respiratory pressure, heart rate and blood pressure on CSF flow in the presence of a spinal CSF obstruction. In animals with a normal SAS, physiological factors did not have a clear effect on spinal CSF flow over the 20 min time course studied or on tracer deposition in the cord. A SAS obstruction, however, was found to decrease spinal CSF flow, and in tachycardic animals this flow was decreased further, albeit transiently (for approximately 15 min). CSF flow was not significantly reduced at 20 min. The combination of hypertension and a SAS obstruction reduced CSF tracer deposition in the spinal cord parenchyma. In the brain parenchyma, hypertension increased CSF tracer influx while positive-pressure ventilation decreased tracer influx. A SAS obstruction reduced tracer deposition in spontaneously breathing animals.

### Cardiorespiratory modulation did not affect spinal CSF flow in the normal SAS

In the normal SAS, manipulation of cardiorespiratory variables did not influence CSF flow in the spine in this study (Supplementary Fig. 4). In contrast to the spinal cord, there were differences in CSF tracer deposition in the brain in spontaneous breathing versus positive-pressure ventilation groups. In animals with a normal SAS, ventilated, normotensive, normocardic animals had reduced CSF tracer influx into the brain parenchyma. This is consistent with previous human imaging studies demonstrating that CSF flow in the cranial subarachnoid compartment is predominantly influenced by breathing and the pulsatile pressures of the cardiac cycle [8, 16–19]. Considering that the anatomical location, vascularization, and ensheathment of the spinal SAS (in a pliable thecal sac) differs greatly from its cranial counterpart, pressures related to cardiac and respiratory function likely have a distinct impact on spinal CSF flow.

The lack of effect of cardiac and ventilatory parameters on spinal CSF flow contrasts with our previous findings, which indicated that the negative intrathoracic pressure generated during spontaneous breathing had a greater effect on spinal CSF flow than arterial pulsations [12]. The differing results may be due to variations in the tidal volume between studies. While Liu et al. [12] used a low tidal volume of 3–4 mL/kg – a volume reported to reduce pulmonary edema in a lung injury model [20] – a tidal volume of ~ 7 mL/kg was used in the present study to reproduce the physiological tidal volume of awake male Sprague-Dawley rats [14]. This volume is reportedly safe and does not cause lung injury [21]. Increasing tidal volume causes a proportional increase in intrathoracic pressure [22]. This means that in the current study ventilated animals likely experienced greater intrathoracic pressure during inhalation, and greater fluctuations in intrathoracic pressure over the respiratory cycle compared to animals in the previous study. If it is the magnitude of change in intrathoracic pressure over the respiratory cycle that drives CSF flow, rather than the shift from negative to positive pressure, then it would be expected that the positive-pressure ventilated rats in our study would have a magnitude of intrathoracic pressure change that more closely resembles that of the spontaneously breathing animals. This might explain the minimal differences in CSF flow between the two groups.

### SAS obstruction substantially reduced spinal CSF flow

Irrespective of the physiological variable modulated, a SAS obstruction markedly reduced CSF flow in the spine. A study by Milhorat et al. [23] investigating the association between Chiari malformation 1 and tethered cord syndrome observed that spinal cord tethering restricted spinal motion in response to respiration and reduced CSF velocity. In the current study a reduction in cord movement during inhalation was observed intraoperatively following the placement of the extradural suture, suggesting that this effectively tethered the cord, which would likely cause a decrease in CSF velocity and may explain the decrease in craniocaudal fluid flow. This is supported by a human imaging study reporting that peak cranial and caudal CSF velocities were reduced in syringomyelia patients compared to healthy controls [24]. This decrease in velocity is likely caused by arachnoiditis at the level of the syrinx, tethering the cord.

### SAS obstruction reduces CSF influx in the brain in spontaneously breathing animals

Surprisingly in spontaneously breathing animals, the spinal SAS obstruction resulted in a reduction in CSF tracer deposition in the brain parenchyma when compared to animals with a normal SAS. This is likely caused by the tethering of the cord at the level of the obstruction and a reduction in CSF velocity. This may have been further exacerbated by the observed decrease in respiratory rate (Supplementary Fig. 1) following the placement of the extradural suture (SAS obstruction). These factors may have prevented the normal shift in CSF from the spinal compartment to the cranium during diastole [25], reducing the CSF tracer deposition in the brain. In the positive-pressure ventilation animals, it is possible that since CSF movement from the spinal compartment to the cranium is already reduced, the addition of the SAS obstruction does not have any noticeable effect.

### Tachycardia reduced spinal CSF flow in the presence of SAS obstruction

Tachycardia in the presence of a SAS obstruction decreased spinal CSF flow for 15 min following CSF tracer injection. Under normal conditions, over the course of the cardiac cycle, blood moves into the cranium in systole, resulting in a corresponding shift of CSF into the spinal compartment to maintain intracranial pressure (in accordance with the Monro-Kellie doctrine). The inverse occurs in diastole, with venous return from the brain, which is counteracted by an influx in CSF from the spinal canal into the cranial compartment [25]. At high heart rates the diastolic portion of the cardiac cycle is shortened [26], preventing the heart from filling with blood completely between contractions, and reducing cardiac output [27]. A decrease in blood flow into the brain would require a smaller compensatory shift in CSF into the spinal compartment in systole, resulting in the observed reduction in spinal CSF flow. After approximately 15 min, the effect of tachycardia in the presence of SAS obstruction was nonsignificant.

### Hypertension affects CSF tracer influx differently in the brain and spinal cord

In animals with a normal SAS, hypertension increased CSF tracer deposition in the brain. Phenylephrine, used in this study to induce hypertension, has been shown to constrict cerebral blood vessels (reviewed in [28]). This would likely result in larger perivascular spaces and facilitate greater movement of CSF into the brain interstitium. The same result wasn’t observed in the spinal cord, potentially because the inverse organization of the spinal cord (white matter surrounding grey matter) with fewer penetrating arterioles directed into the grey matter [29, 30] minimizes the impact of altered periarterial space flow on net CSF movement.

It was only in the presence of a SAS obstruction that hypertension induced a significant decrease in tracer influx into the cord. Intraoperatively, it was evident that the extradural constriction effectively narrowed the SAS and tethered the cord, and likely reducing CSF velocity. This reduced the craniocaudal flow of CSF under all physiological conditions, but did not translate to reduced tracer influx into the spinal cord except in hypertensive animals. In a pig model of SCI, Bessen et al. [31] found that mean CSF velocity was reduced above and below the injury site during the acute injury stage when cord swelling completely obstructed the SAS. The hypertensive animals with SAS obstruction had reduced arterial oxygenation (compared to other experimental groups, Supplementary Fig. 2F), which would induce vasodilation to increase blood flow and overcome the oxygen deficit [32]. The vasodilation would reduce perivascular space size and further reduce CSF flow into the spinal cord [33].

### Positive-pressure ventilation affects CSF influx in the brain

In contrast to the spinal cord, there were differences in CSF tracer deposition in the brain in spontaneous breathing versus positive-pressure ventilation groups. In animals with a normal SAS, ventilated, normotensive, normocardic animals had reduced CSF tracer influx in the brain parenchyma. Respiration can be classified as abdominal or thoracic breathing. Movement of the diaphragm is responsible for abdominal breathing, which has been found to produce higher CSF flow rates and volumes than thoracic breathing [34]. Positive-pressure ventilated animals were administered a neuromuscular blockade to paralyse the diaphragm, preventing animal/ventilator dyssynchrony. This effectively removes the negative intrathoracic pressure component of the respiratory cycle usually generated by the downward movement of the diaphragm during inspiration, and likely led to reduced cranial movement of CSF.

### Hypercapnia and hyperoxia: implications for interpretation

A limitation of this study was the development of hypercapnia and hyperoxia in all animals (Supplementary Fig. 2) compared to normative physiological values under anesthesia [35] and awake [36]. Both hypercapnia and hyperoxia are known to influence cerebrovascular tone [37, 38]. Hypercapnia is a potent vasodilator, increasing cerebral blood flow and volume, which in turn displaces CSF from the cranium, while hyperoxia leads to vasoconstriction and reduced cranial blood flow and volume, drawing CSF from the spinal canal to the cranium [39–42]. It should be noted that these conditions may have influenced the CSF flow dynamics measured in this study. Similarly, isoflurane anesthesia can negatively impact CSF circulation [43], depress respiratory action and increase the hypercapnic state [44]. However, as the gross effects of hypercapnia and hyperoxia are opposite, and inhalation anesthesia was used for all animals with a consistent respiratory rate, it may be the case that the effects on CSF flow are counteracted or equal. Future studies using normocapnic, normoxic conditions would help to clarify how these cardiorespiratory factors influence CSF dynamics.

### Clinical implications

The results of this study demonstrate the complexity of CSF circulation and the numerous factors that influence flow in the central nervous system. The study highlights how pathology not only disrupts CSF flow, but changes how blood pressure, heart rate and breathing influence flow. This is especially relevant in conditions such as PTS where patients often experience dysregulated blood pressure, with bouts of low blood pressure (orthostatic hypotension), episodes of high blood pressure (autonomic dysreflexia), have impaired diaphragm function, reduced lung volumes, and in the case of high cervical injuries, periods of mechanical ventilation [45].

Since hypertension combined with a SAS obstruction reduced CSF influx into the spinal cord in this study, it is likely that hypotension would have an inverse effect. In this instance, it might prove clinically important to treat orthostatic hypotension promptly following spinal trauma to prevent further accumulation of CSF in the cord and the development of PTS. Future studies investigating if there is a correlation between orthostatic hypotension and PTS are warranted.

## Conclusions

This study reported the complicated interactions between cardiorespiratory parameters and CSF movement in the presence of a spinal CSF obstruction. While the modulation of cardiovascular and respiratory parameters had little effect on CSF flow in the normal spinal cord, in the presence of an obstruction in the SAS, spinal subarachnoid CSF flow was significantly lower. In the presence of SAS obstruction, hypertension reduced CSF tracer deposition in the spinal interstitium, which has potential implications for people with blood pressure dysregulation from SCI and their risk of developing PTS. Further investigation is required.

## Supplementary Information

Below is the link to the electronic supplementary material.

Supplementary Material 1

## Data Availability

The datasets used and/or analysed during the current study are available from the corresponding author on reasonable request.

## Abbreviations

ANOVA: Analysis of variance
AU: Arbitrary units
CO_2_: Carbon dioxide
CSF: Cerebrospinal fluid
ICG: Indocyanine green
MAP: Mean arterial pressure
MRI: Magnetic resonance imaging
O_2_: Oxygen
OA-647: Ovalbumin Alexa Fluor-647 conjugate
PaCO_2_: Partial pressure of carbon dioxide
PaO_2_: Partial pressure of oxygen
PBS: Phosphate buffered saline
PFA: Paraformaldehyde
PTS: Post-traumatic syringomyelia
SAS: Spinal subarachnoid space
SCI: Spinal cord injury
SD: Standard deviation
