# Supplementary material for: Cardiorespiratory modulation of cerebrospinal fluid flow in the presence of spinal subarachnoid space obstruction
Source: Fluids Barriers CNS. 2026 Jun 9;23:90. doi: 10.1186/s12987-026-00823-4 (PMC13403395; doi:10.1186/s12987-026-00823-4)
Supplement: Supplementary file 1 — Supplementary Material 1 [file 12987_2026_823_MOESM1_ESM.docx]

**
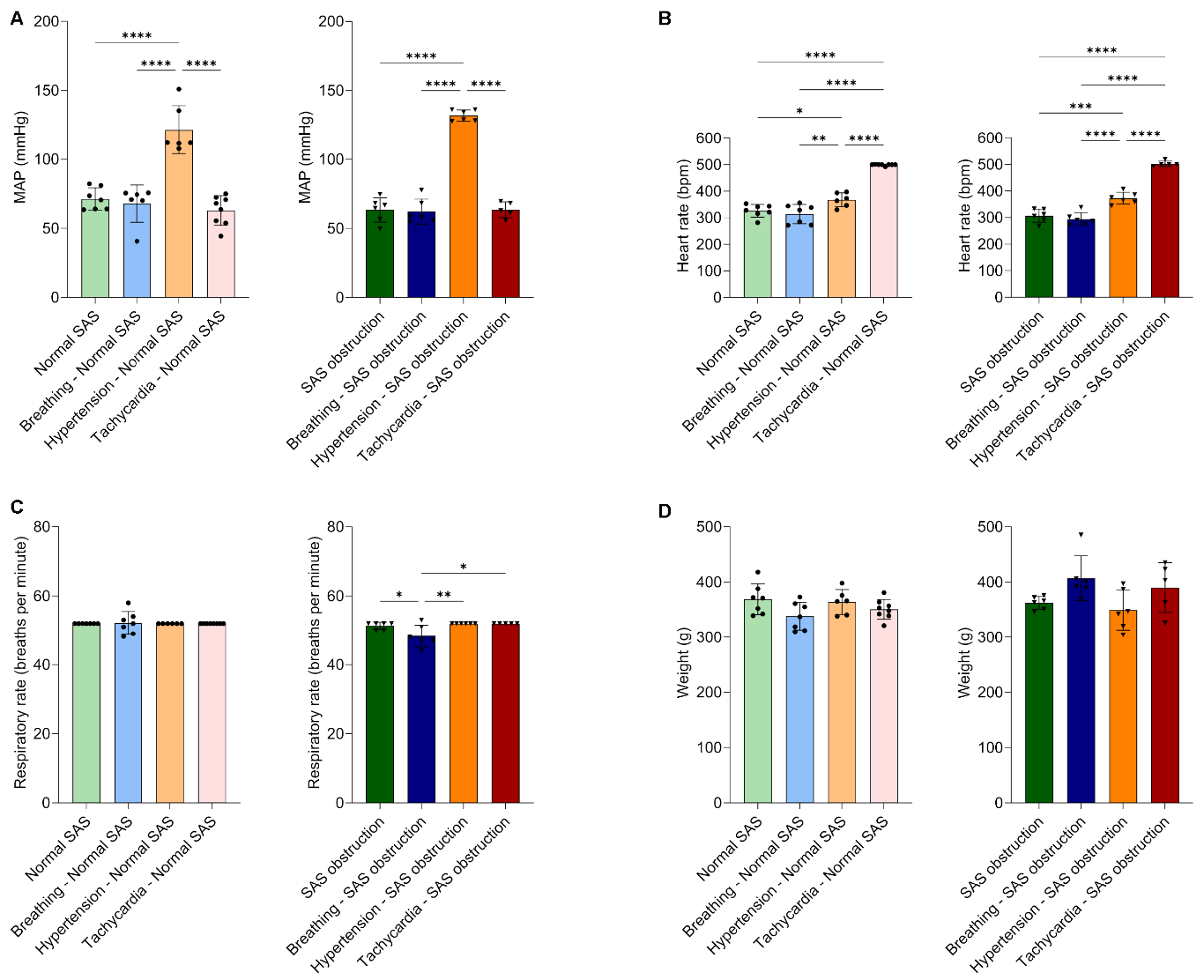
**

**Supplementary Figure 1: Modulation of physiological variables in animals with a normal or obstructed subarachnoid space (SAS).** Animals received an intracisternal injection of cerebrospinal fluid (CSF) tracer. The CSF tracer circulated for 20 min, during which time physiological variables were carefully monitored using pulse oximetry, and via an arterial catheter connected to a pressure transducer. The following variables were recorded: (**A**) mean arterial pressure (MAP), (**B**) heart rate, (**C**) respiratory rate, and (**D**) weight. (**A**) Hypertensive animals received an infusion of phenylephrine via the femoral vein which significantly increased MAP compared to all other animal groups. (**B**) Cardiac stimulation significantly increased heart rate of tachycardic animals (a difference of >100 bpm) compared to all other groups. Phenylephrine increased heart rate, and the hypertensive animals had a higher heart rate, but this increase was still significantly lower than the tachycardic group (mean ± SD). (**C**) Respiratory rate was recorded in the spontaneous breathing groups. All other animal groups were positive-pressure ventilated at 50 – 52 breaths per min. There was no difference in respiratory rate between animals with a normal SAS. The SAS obstruction caused a decrease in breathing rate, resulting in a significant difference in the spontaneous breathing group. (**D**) There was no difference in animal weights between groups. The results are presented as mean ± SD (n = 5 – 8), *p ≤ 0.05, **p ≤ 0.01, ***p ≤ 0.001, ****p ≤ 0.0001; one-way ANOVA + post hoc Bonferroni test.


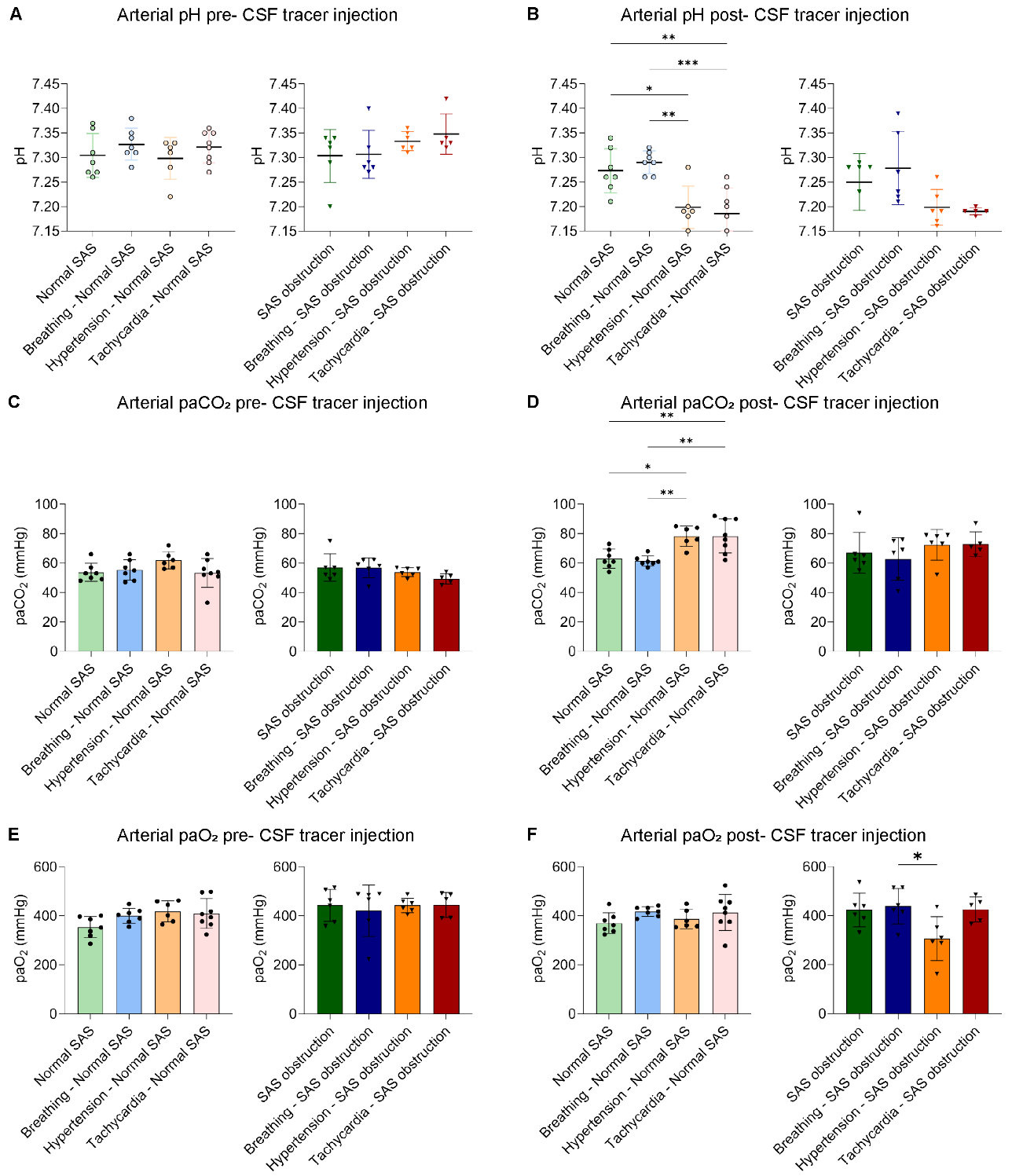


**Supplementary Figure 2:** **Arterial blood gas analysis of animals with a normal or obstructed subarachnoid space (SAS).** Animals received an intracisternal injection of cerebrospinal fluid (CSF) tracer. The CSF tracer circulated for 20 min. Arterial blood gas analysis was performed immediately prior to CSF tracer injection, following the modulation of the physiological variable being tested: respiratory pressure (spontaneous breathing), blood pressure (hypertension), or heart rate (tachycardia) (**A**, **C**, **E**), and at the end of the experiment (**B**, **D**, **F**). The following measurements were compared between animal groups: (**A**, **B**) arterial blood pH, (**C**, **D**) partial pressure of carbon dioxide (paCO_2_), and (**E**, **F**) partial pressure of oxygen (paCO_2_). The results are presented as mean ± SD (n = 5 – 8), *p ≤ 0.05, **p ≤ 0.01, ***p ≤ 0.001; one-way ANOVA + post hoc Bonferroni test.


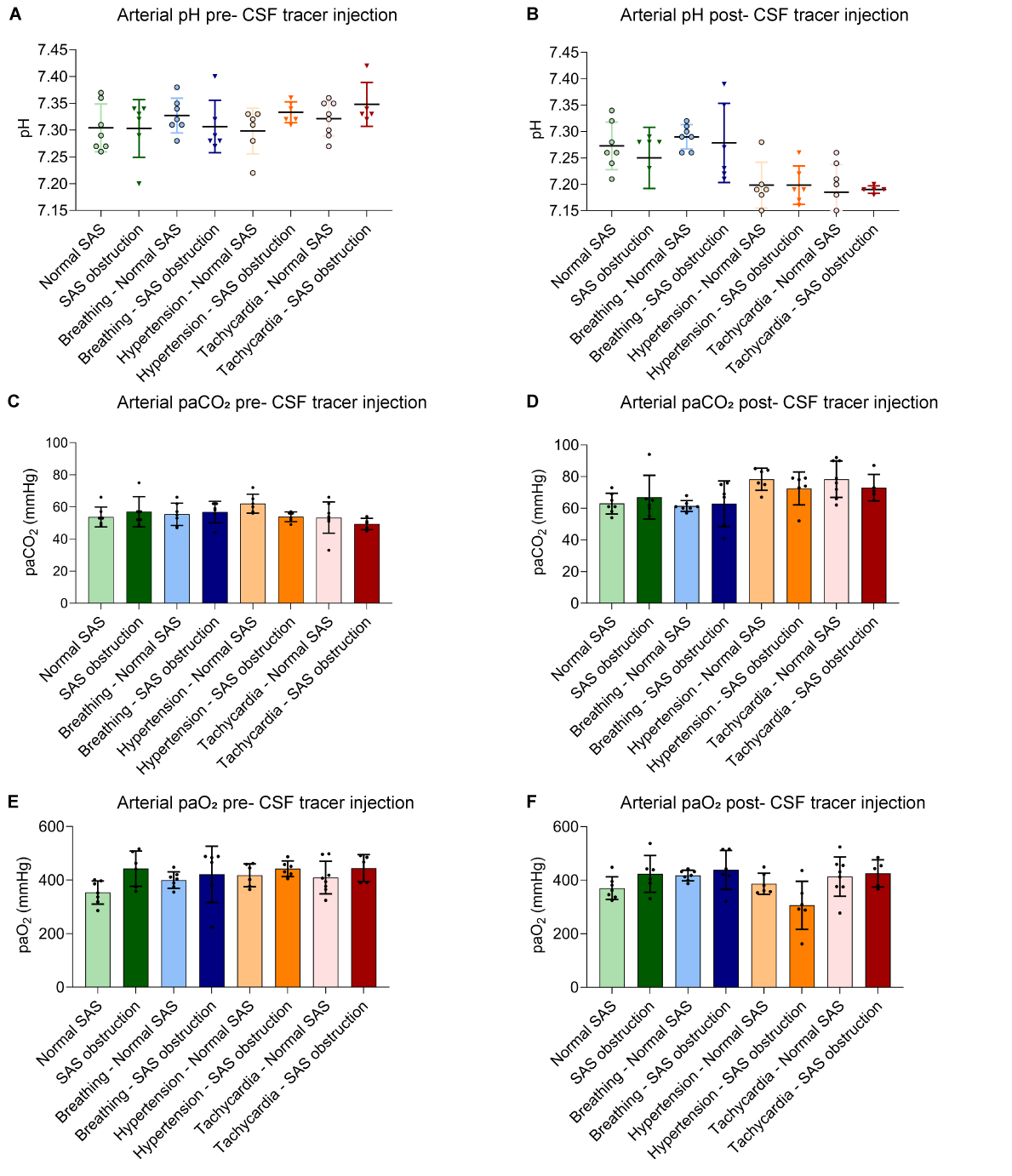


**Supplementary Figure 3:** **Arterial blood gas analysis comparing animals with a normal or obstructed subarachnoid space (SAS).** Animals received an intracisternal injection of cerebrospinal fluid (CSF) tracer. The CSF tracer circulated for 20 min. Arterial blood gas analysis was performed immediately prior to CSF tracer injection, following the modulation of the physiological variable being tested: respiratory pressure (spontaneous breathing), blood pressure (hypertension), or heart rate (tachycardia) (**A**, **C**, **E**), and at the end of the experiment (**B**, **D**, **F**). The following measurements compared normal and SAS obstruction groups based on the modulated physiological variable: (**A**, **B**) arterial blood pH, (**C**, **D**) partial pressure of carbon dioxide (paCO_2_), and (**E**, **F**) partial pressure of oxygen (paCO_2_). The results are presented as mean ± SD (n = 5 – 8); one-way ANOVA + post hoc Bonferroni test.


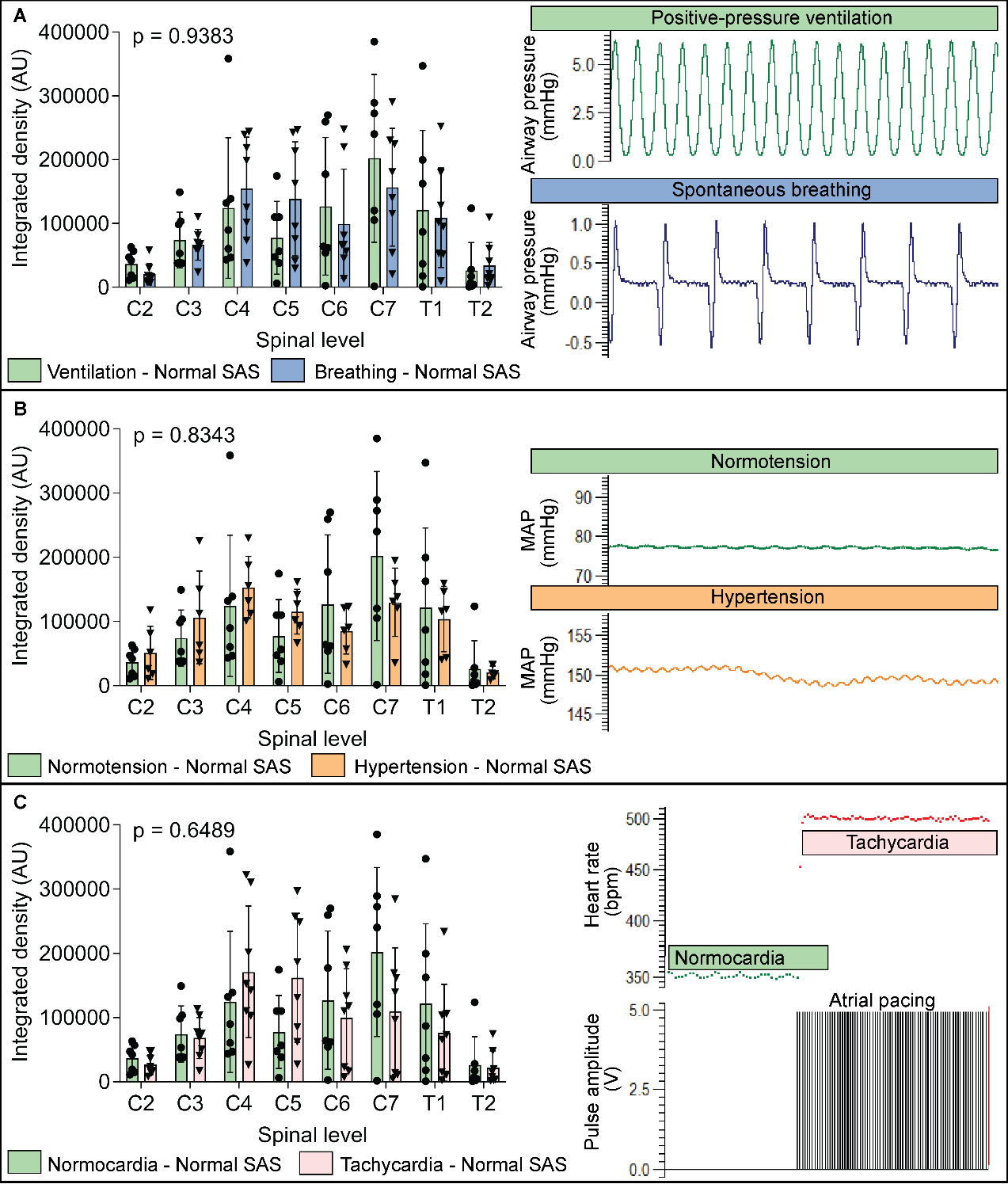


**Supplementary Figure 4: Modulation of physiological variables did not affect spinal cerebrospinal fluid (CSF) flow.** There was no significant difference in CSF tracer movement in the normal subarachnoid space (SAS) of (**A**) spontaneous breathing animals, (**B**) hypertensive animals, and (**C**) tachycardic animals compared to animals that were positive-pressure ventilated, normotensive, and normocardic. Graphs show fluorescence intensity of spinal levels at 20 min following tracer injection. The results are presented as mean integrated density ± SD (n = 6 – 8), one-way ANOVA + post hoc Bonferroni test.


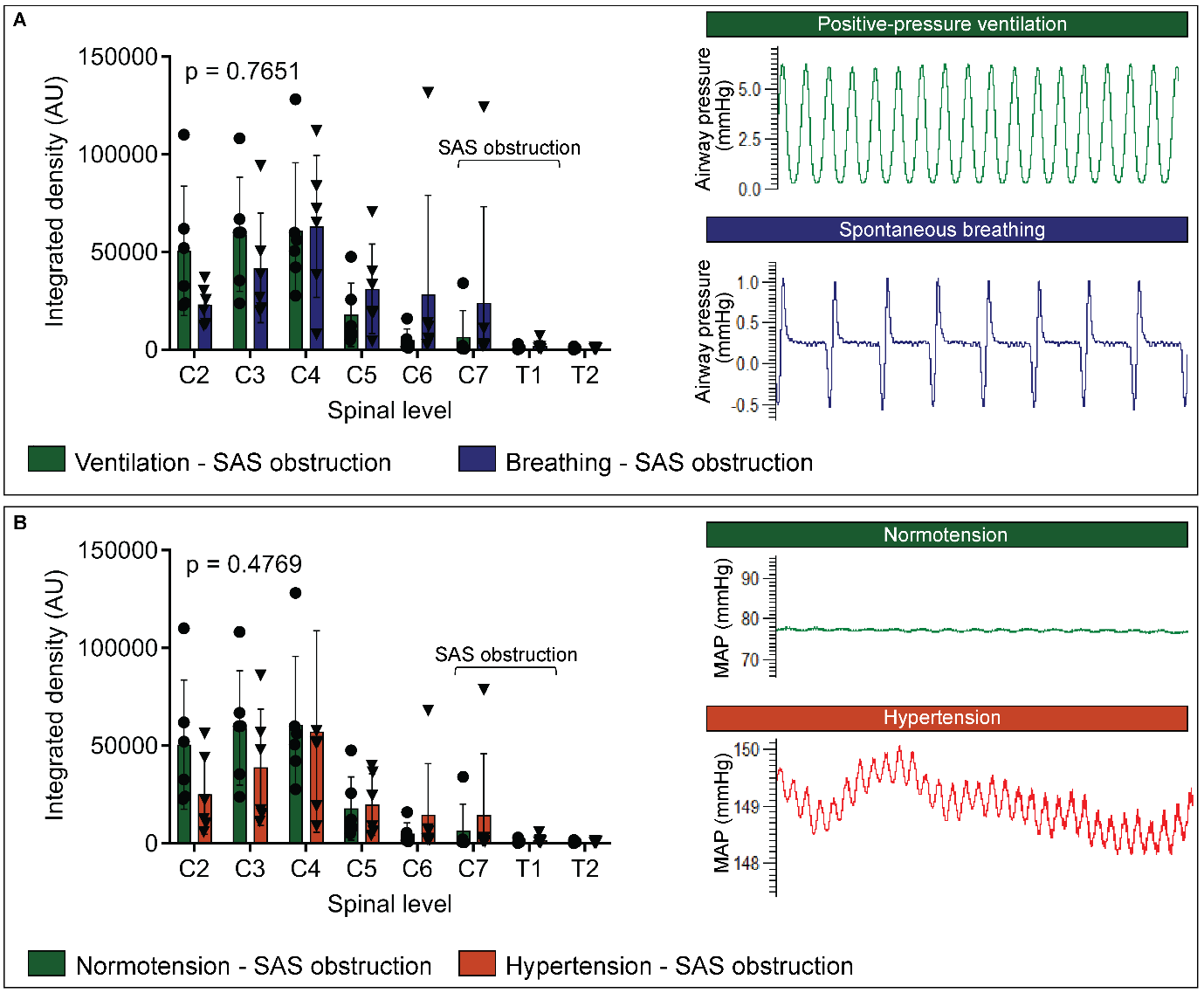


**Supplementary Figure 5: Respiration and hypertension did not alter spinal cerebsrospinal fluid (CSF) flow when CSF was obstructed.** In animals with a SAS obstruction there were no significant differences in CSF tracer flow in the spinal SAS of positive-pressure ventilated, normotensive animals compared to: (**A**) spontaneous breathing animals, or (**B**) hypertensive animals. Graphs show fluorescence intensity of spinal levels at 20 min following tracer injection. The results are presented as mean integrated density ± SD (n = 6), two-way ANOVA + post hoc Bonferroni test.


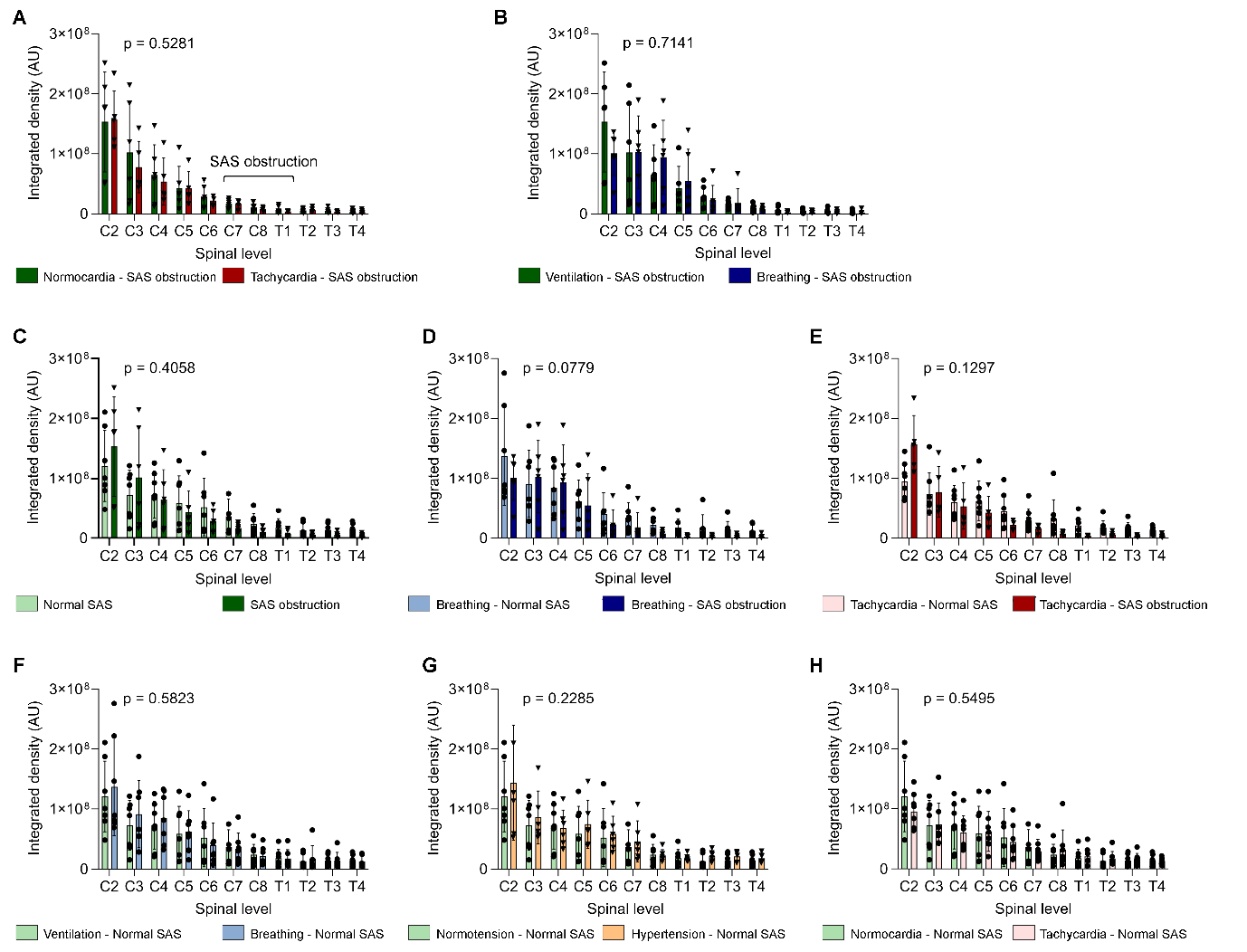


**Supplementary Figure 6: Effect of respiration, hypertension, and tachycardia on cerebrospinal fluid (CSF) tracer flow into the cord.** (**A**, **B**) In animals with a SAS obstruction there was no significant difference in CSF tracer influx in the spinal cord of: (**A**) tachycardic animals, or (**B**) spontaneous breathing animals, compared to positive-pressure ventilated, normocardic animals. (**C** – **E**) A SAS obstruction did not result in a significant difference in CSF tracer influx in the spinal cord of (**C**) positive-pressure ventilated, normotensive, normocardic animals, (**D**) spontaneous breathing animals, and (**E**) tachycardic animals. (**F** – **H**) In animals with a normal SAS: (**F**) breathing, (**G**) hypertension, and (**H**) tachycardia, did not result in a significant change in CSF tracer influx in the spinal cord. Graphs show fluorescence intensity of spinal cord levels at 20 min following tracer injection. The results are presented as mean integrated density ± SD (n = 5 – 8), two-way ANOVA + post hoc Bonferroni test.


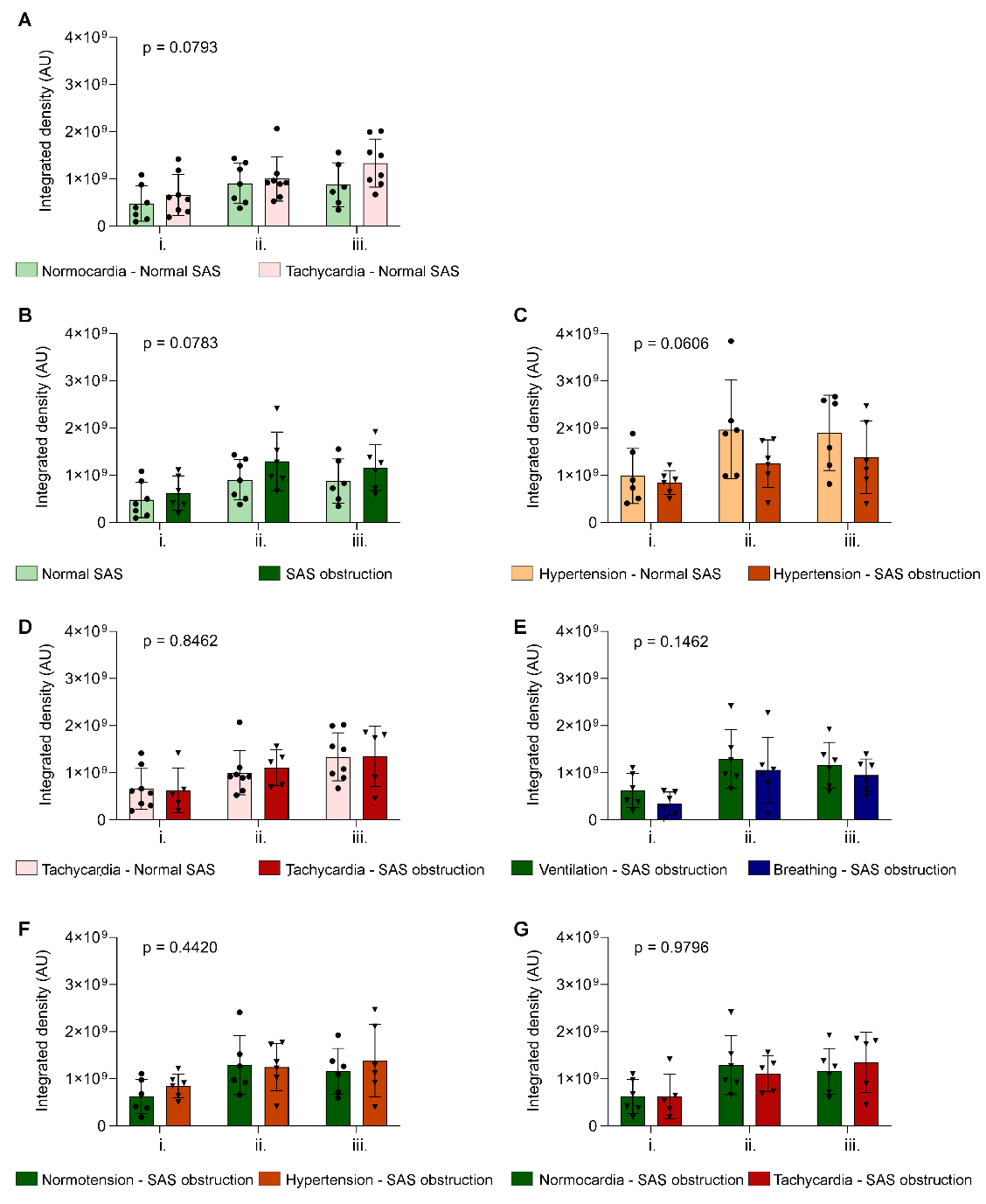


**Supplementary Figure 7: Effect of respiration, hypertension, and tachycardia on cerebrospinal fluid (CSF) tracer flow into the brain.** (**A**) In animals with a normal SAS, tachycardia did not significantly alter CSF tracer influx into the brain. (**B** – **D**) A subarachnoid space (SAS) obstruction did not result in a significant difference in CSF tracer influx in the brain of (**B**) positive-pressure ventilated, normocardic, normotensive animals, (**C**) hypertensive animals, and (**D**) tachycardic animals. (**E** – **G**) In animals with a SAS obstruction there was no significant difference in CSF tracer influx in the anterior (i), thalamic (ii), and cerebellar (iii) brain regions when (**E**) respiratory pressure, (**F**) blood pressure, or (**G**) heart rate were altered. Graphs show fluorescence intensity of brain regions at 20 min following tracer injection. The results are presented as mean integrated density ± SD (n = 5 – 8), two-way ANOVA + post hoc Bonferroni test
